# Clinical comparison of transfemoral vs. distal transradial access for lower extremity arteriography

**DOI:** 10.3389/fsurg.2025.1677953

**Published:** 2026-01-12

**Authors:** Zibo Feng, Qionglin Zeng, Youpeng Zhu, Ting-yu Wang

**Affiliations:** Department of Wound Repair Surgery, Liyuan Hospital, Tongji Medical College, Huazhong University of Science and Technology, Wuhan, Hubei, China

**Keywords:** anatomical snuffbox, distal transradial access (dTRA), lower extremity angiography, lower extremity ulcer, transfemoral access (TFA)

## Abstract

**Objectives:**

This study aims to evaluate the feasibility and advantages of the distal transradial approach (dTRA) for assessing blood supply in patients with lower limb ulcers.

**Methods:**

This study analyzed 66 patients with chronic lower limb ulcers who underwent lower limb angiography between December 2023 and October 2024. Among them, 44 patients were in the transfemoral access (TFA) group, and 22 patients were in the dTRA group. The success rate of the procedure, contrast dosage, time consumed for contrast, postoperative complications, patient Satisfaction Score and objective analysis of image quality were recorded and statistically analysed using SPSS 22.0.

**Results:**

The analysis revealed that there was no statistically significant difference in the procedural success rates between the two groups, with the TFA group achieving a 95% success rate compared to 86% in the dTRA group (*P* = 0.41). The amount of contrast agent used was significantly higher in the dTRA group than in the TFA group (70.79 ± 13.25 mL vs. 60.10 ± 16.98 mL, *P* = 0.02). However, there was no statistically significant difference in the complication rate (2.3% in the TFA group vs. 0% in the dTRA group, *P* = 1.00). Slightly more time-consuming imaging in the dTRA group (30 min vs. 25 min, *P* = 0.06). Patient satisfaction score in the dTRA group (4.16 ± 0.60) was better than the TFA group (3.12 ± 0.63, *p* = 0.00). The CNR (13.99 ± 7.92) in the dTRA group was lower than that in the TFA group (21.31 ± 10.57) (*P* = 0.009).

**Conclusions:**

The dTRA demonstrates diagnostic efficacy comparable to the TFA in evaluating lower limb blood supply in chronic lower limb ulcers patients, and significantly improves patient comfort with its minimally invasive access, early mobility, and low complication risk.

## Introduction

Although there are many methods for imaging diagnosis of lower extremity arterial disease, lower extremity arteriography is still the gold standard and plays a key role in interventional diagnosis and treatment (1, 2). The transfemoral approach remains the most widely adopted standard access for lower extremity arteriography at the present time due to its direct anatomical pathway, well-established techniques, and procedural convenience (3). However, the transfemoral approach presents significant limitations in situations such as absent femoral pulse and recent arterial surgery with fresh scars in the groin, along with the requirement for prolonged post-procedural limb immobilization which is often poorly tolerated by patients (4). Currently, numerous researchers are actively exploring the application of distal transradial access for the diagnosis and treatment of lower extremity arterial disease (5). Distal transradial approach punctures the distal radial artery through the region of the hand's anatomical snuffbox, making full use of the dual blood supply in the palm of the hand (rich in anastomotic branches of the radial and ulnar arteries) and significantly reducing the risk of occlusion, spasm, and other complications of the radial artery caused by traditional radial artery access (6–8). Additionally, dTRA eliminates the need for strict post-procedural immobilization, significantly enhancing patient comfort and early mobility. These technical advantages, coupled with the continuously optimized adaptability of interventional devices, make dTRA show the potential to replace the TFA approach. Although the dTRA approach has been explored for lower extremity angiography, there are still few feasibility studies on patients with lower extremity ulcers (especially patients with diabetic foot), and the relevant data are relatively limited. This study systematically compares the clinical outcomes of transfemoral approach with dTRA for lower limb arteriography, focus on procedural success, post-interventional complications, and patient comfort level, in order to provide a more comprehensive reference for clinical decision-making.

## Method

### Study procedures

All patients with chronic ulcers of the lower extremities (regardless of age and gender) who underwent arteriography of the lower extremities were included in this study. The clinical research protocol complied with the ethical principles of the Declaration of Helsinki (1975) and was approved by the Institutional Medical Ethics Committee of our hospital (Ethics Approval No: IRB ID: [2021]IEC(YJ002). Written informed consent was obtained from all participants prior to enrollment.

### Study design and population

This single-center retrospective observational study consecutively enrolled 66 patients who underwent lower extremity arterial angiography at Liyuan Hospital, Tongji Medical College, Huazhong University of Science and Technology between December 2023 and October 2024. The cohort primarily comprised patients with diabetic foot, including a subset with simple chronic lower limb ulcers. According to the vascular access approach used, patients were divided into two groups: the transfemoral artery (TFA) group (*n* = 44) and the distal transradial artery (dTRA) group (*n* = 22).

The grouping was based on real-world clinical practice during the same period, wherein two medical teams operated in parallel at the center. In one team, a physician proficient in both distal transradial and femoral artery puncture selected the access approach based on comprehensive clinical assessment and patient preference—for example, opting for dTRA if the patient wished to avoid prolonged post-procedural immobilization. The other team consistently used the transfemoral approach. This naturally formed grouping mode realized the relative comparability of the baseline data of the two groups without imposing additional selection criteria, and all patients who met the inclusion criteria were included in the analysis.

### Study procedures

#### Transfemoral arteriography

The patient was placed in the supine position, and the inguinal region was exposed. After local anaesthesia with lidocaine, arterial access was performed using a modified Seldinger technique with the insertion of a 4F arterial sheath. Angiography of the puncture site was first performed. Under fluoroscopic guidance, the guidewire was advanced with the support of a selective angiographic catheter, crossing the terminal aorta and entering the contralateral common femoral artery. After removing the guidewire, angiography is performed. After imaging is complete, exit the guidewire and remove the sheath. Apply pressure to the puncture site and wrap it for 6–8 h (Table 1).

**Table 1:** Intraoperative use of instruments.

Intraoperative instruments	Manufacturer and specifications
Micropuncture	COOK (21G, 7 cm)
Radifocus introducer II	TERUMO (4F)
Guidewire	Terumo RF*GA35153M
Angiographic Catheter	Cordies (4F, 100 cm)

#### Distal transradial arteriography

The patient was placed in the supine position with the arm at the side. The hand and forearm were routinely sterilised and toweled. Local anaesthesia (2% lidocaine) was administered at the anatomical snuffbox, followed by ultrasound-guided puncture of the distal radial artery using a minipuncture needle and a modified Seldinger technique. A 5F radial artery sheath was placed. Under digital subtraction angiography (DSA), contrast was injected to confirm patency of the radial and brachial arteries. The angiographic catheter was then superselected to the bilateral external iliac arteries for angiography. Upon completion, the catheters and sheaths were removed and a pressure dressing was applied to the anatomical snuffbox for 2 h (Table 2).

**Table 2:** Intraoperative use of instruments.

Intraoperative instruments	Manufacturer and specifications
Puncture needle	COOK (SDN-17.8-7.0)
Braidin vascular sheath group	APTMedical (5F)
Guidewire	Terumo RF*GA35263M
Hydropointer hydrophilic coated contrast catheter	APTMedical (5F, 130 cm)

### Study endpoints and definitions

The primary endpoint was the surgical success rate, defined as the probability of successfully completing angiography as planned to achieve the diagnostic objective for lower extremity arterial disease. Secondary endpoints included contrast medium dosage, contrast procedure duration, complication rates, patient satisfaction score, and objective image quality assessment. Patient satisfaction score was rated using a 5-point Likert scale (9) (1 for very dissatisfied, 2 for less satisfied, 3 for generally satisfied, 4 for more satisfied, and 5 for very satisfied). The objective analysis index of image quality uses signal-to-noise ratio(SNR) and contrast-to-noise ratio(CNR) to perform objective quantitative analysis of the vessels at the trifurcation of the inferior genicular artery (10, 11). (SNR = S_vessel/SD_background, S_vessel is the average signal intensity of blood vessels, and SD_background is the standard deviation of background noise. CNR = |S_vessel—S_background|/SD_background, S_background is the average signal strength of the adjacent background area).

### Statistical analysis

Statistical analysis was performed using SPSS 22.0 software. Normally distributed measurement data were expressed as mean ± standard deviation (X ± s) and compared using independent samples *t*-test between groups. Non-normally distributed measurement data were presented as median (interquartile range) [M (P25, P75)] and analyzed using Wilcoxon signed-rank test for intergroup comparisons. Categorical data were expressed as percentages (%) and compared using chi-square (X^2^) test. A *P*-value < 0.05 was considered statistically significant.

## Result

### Baseline characteristics

No significant difference was observed in gender distribution between the dTRA group (*n* = 22) and TFA group (*n* = 44), the proportion of males in both groups was 72.7% (*P* = 1.00). Regarding age, the dTRA group had a mean age of 71.45 ± 10.85 years, compared to 65.20 ± 12.96 years in the TFA group. The between-group difference approached but did not reach statistical significance by independent samples *t*-test (*p* = 0.06). In terms of underlying diseases, the prevalence of hypertension was 68.2% in dTRA group and 72.7% in TFA group (*P* = 0.70), the prevalence of coronary heart disease was 45.5% vs. 50.0% (*P* = 0.73), and the prevalence of type 2 diabetes mellitus was 90.9% and 86.4% (*P* = 0.59). And there was no significant difference in distribution of the various types of underlying diseases between the two groups (*P* > 0.05). These results indicate that the two groups of patients were well comparable in terms of baseline characteristics (Table 3).

**Table 3:** Comparison of baseline characteristics between two groups.

Patient Characteristics	dTRA	TFA	*P*-value
Age (years)	71.45 ± 10.85	65.20 ± 12.96	0.06
Male, *n* (%)	16 (72.7)	32 (72.7)	1.00
Hypertension, *n* (%)	15 (68.2)	32 (72.7)	0.70
Coronary Artery Disease, *n* (%)	10 (45.5)	22 (50.0)	0.73
Type 2 Diabetes Mellitus, *n* (%)	20 (90.9)	38 (86.4)	0.59

### Surgical success rate

**Figure 1:**
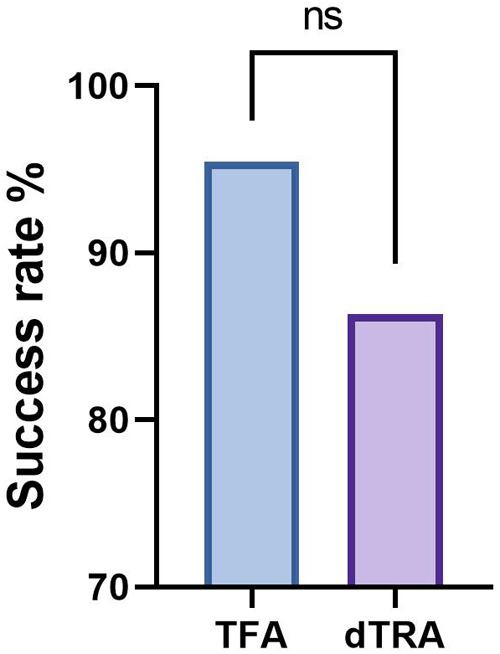
Surgical success rate success rates between two groups.

The dTRA group succeeded in 19 cases and failed in 3 cases (86%). While the TFA group had 42 successful cases and 2 failures (95%). There was no significant difference in surgical success rates between the two groups by chi-square test (X^2^ = 0.68, *P* = 0.41) (Table 4, Figure 1).

**Table 4:** Comparison of procedural success rates between two groups.

Group	Cases (*n*)	Success	Failure
dTRA	22	19	3
TFA	44	42	2
Test statistic	*c*^2^ = 0.68		
*P*-value	0.41		

### Contrast medium volume

**Figure 2:**
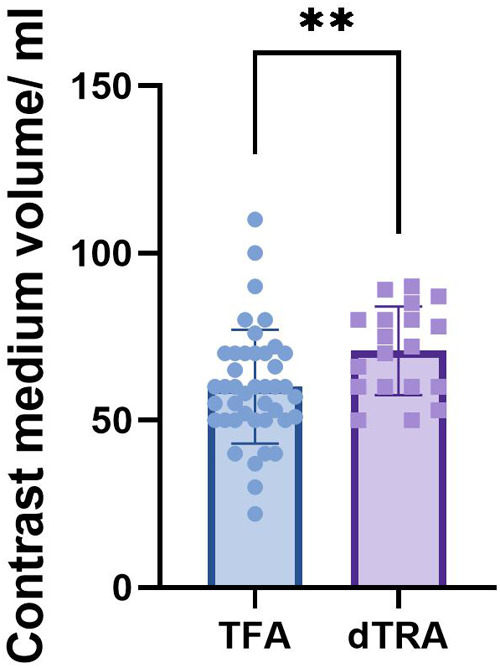
Contrast medium volume between two groups.

The dTRA group required significantly more contrast medium (70.79 ± 13.25 mL) compared to TFA group (60.10 ± 16.98 mL). Independent samples t-test confirmed this statistically significant difference (*t* = 2.43, *P* = 0.02) (Table 5, Figure 2).

**Table 5:** Comparison of contrast medium volume between two groups.

Group	Cases (*n*)	Contrast Medium Volume
dTRA	19	70.79 ± 13.25
TFA	42	60.10 ± 16.98
Test statistic	*t* = 2.43	
*P*-value	0.02	

### Complication rates

In the TFA group, there was 1 case of pseudoaneurysm as a complication. In the dTRA group, all 19 patients had no complications.There was no significant difference in surgical success rates between the two groups by chi-square test (*χ*^2^ = 0.00, *P* = 1.00) (Table 6).

**Table 6:** Comparison of complication rates between the two groups.

Group	Cases (*n*)	Complication-free	With complications
dTRA	19	19	0
TFA	42	41	1
Test statistic	*c*^2^ = 0.00		
*P*-value	1.00		

### Contrast procedure duration

**Figure 3:**
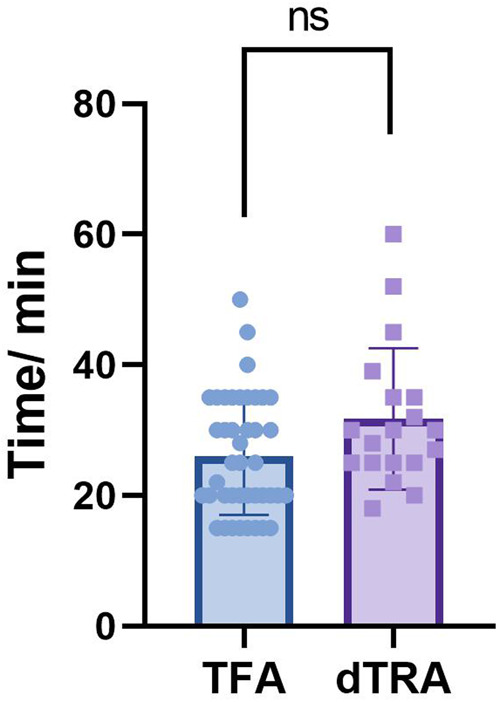
Contrast procedure duration between two groups.

The median procedure time was 30 (25–35) minutes for the dTRA group vs. 25 (20–35) minutes for the TFA group. Wilcoxon signed-rank test showed no statistically significant difference between groups (Z = 1.86, *P* = 0.06) (Table 7, Figure 3).

**Table 7:** Comparison of procedure time between the two groups.

Group	Cases (*n*)	Procedure Time M (P25, P75)
dTRA	19	30 (25–35)
TFA	42	25 (20–35)
Test statistic	Z = 1.86	
*P*-value	0.06	

### Patient satisfaction score

**Figure 4:**
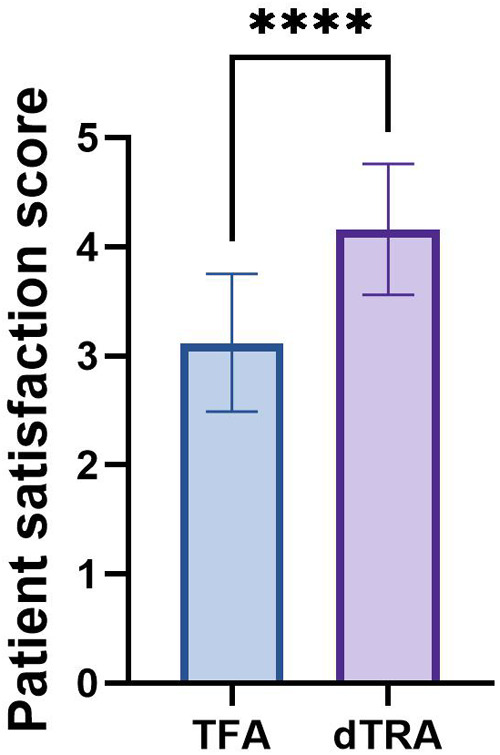
Patient satisfaction score between two groups.

Patient satisfaction scores were (4.16 ± 0.60) in the dTRA group and (3.12 ± 0.63) in TFA group. Statistically analysed, there was a significant difference in patient satisfaction scores between the two groups (*t* = 6.03, *P* = 0.00) (Table 8, Figure 4).

**Table 8:** Comparison of patient satisfaction score between the two groups.

Group	Cases (*n*)	Patient Satisfaction Score
dTRA	19	4.16 ± 0.60
TFA	42	3.12 ± 0.63
Test statistic	*t* = 6.03	
*P*-value	0.00	

### Objective image quality assessment

In the dTRA group, CNR was (13.99 ± 7.92), SNR was (77.37 ± 29.23); The CNR and SNR of the TFA group were (21.31 ± 10.57) and (70.37 ± 23.21), respectively. Statistical analysis showed that the CNR score of the transfemoral puncture group was significantly different (*t* = 2.69, *P* = 0.009) (Table 9).

**Table 9:** Comparison of CNR and SNR scores between the two groups.

Group	Cases (*n*)	CNR	SNR
dTRA	19	13.99 ± 7.92	77.37 ± 29.23
TFA	41	21.31 ± 10.57	70.37 ± 23.21
Test statistic		*t* = 2.69	*t* = 0.953
*P*-value		0.009	0.344

## Disscusion

In cardiac interventions, dTRA is emerging as the preferred route, as its benefits are reflected in a reduced complication rate with increased patient comfort (5, 12–15). In order to accurately assess the blood supply of lower limbs of patients with diabetes foot and provide personalized treatment plans, the center carried out special research. The results showed that distal radial artery access (dTRA) demonstrated significant clinical potential in lower limb arteriography, opening up new pathways for diabetic foot diagnosis and treatment.

In terms of surgical success rate, the TFA group had a slightly higher surgical success rate than the dTRA group, and the difference was not statistically significant. This suggests that dTRA demonstrates comparable clinical feasibility to TFA in lower limb arteriography. With the inherent advantages of a direct anatomical path, a mature technical system, and a standardised procedure, TFA has demonstrated remarkable convenience in clinical application. For example, due to the extensive clinical experience of the operators, the success rate of this procedure is almost 100% In our centre. The 2 failures in this study were due to obstruction of anatomical access due to occlusion of the iliac arteries and not due to limitations of the technique itself. By contrast, dTRA requires more skill from the operator due to the anatomical characteristics of the target vessel's thinner diameter (average 2.0–2.5 mm) (15) and tortuous travel, and there is a significant learning curve effect (16, 17). Based on the original intention of improving patients' medical experience, our centre has actively explored the technique of distal radial artery puncture angiography. In practice, it has been found that, with the gradual accumulation of the operator's experience in the operation, the success rate of this procedure has been significantly improved, and the speed of puncture has become faster. Surgical success rates for dTRA have improved significantly with operator experience. Current evidence suggests that the level of technical proficiency stabilizes after about 150 cases (17, 18), and procedural success rates of 90%–99% for skilled practitioners (19–23). Furthermore, a small number of studies have successfully employed dTRA to treat diseases of the iliac artery and superficial femoral artery (24, 25).The data from all these studies fully confirm the technical feasibility of dTRA in the field of lower limb vascular interventions from the technique.

In terms of contrast volume, the dTRA group required a significantly higher mean contrast volume of 70.79 mL compared to the 60.10 mL of the TFA group. This may be due to the relatively tortuous vascular path from the distal radial artery to the arteries of the lower limbs (26), which requires more contrast to obtain a clear vascular image during catheter over-selection and contrast diffusion. Secondly, the lack of operational experience in the early stages of the application of the technique of dTRA in our centre may have led to a less than optimal strategy for contrast administration, thus increasing the overall dosage. Although there is currently no significant difference in complication rates between the two groups, and no cases of contrast-induced nephropathy have occurred, the higher contrast agent dosage may increase potential risks such as renal burden (27). We need to reduce the amount of contrast medium as much as possible while ensuring the quality and safety of the imaging. Only one patient in transfemoral approach developed a pseudoaneurysm after puncture among all patients, which was successfully resolved with ultrasound-guided thrombin injection without sequelae. This observation aligns with published literature reporting that the incidence of pseudo aneurysms associated with the transfemoral approach ranges from 0.05 to 6% (28). Complications are often difficult to avoid in some patients because of obesity and poor postoperative braking. The dTRA offers significant clinical advantages in minimising access site bleeding and vascular complications, including reduced incidence of radial artery occlusion, arterial injury and vasospasm, due to its unique anatomical advantages (29–31). It should be noted that the failure to observe the above related complications in patients of dTRA is most likely due to the limited sample size of this study.

The study found no significant difference in median procedure time between the two groups. The dTRA exhibited longer imaging time (30 min, 25–35) compared to the TFA (25 min, 20–35). This phenomenon may be related to the complex vascular alignment of the dTRA and the increased difficulty of catheter over-selection. Notably, with the accumulation of operator experience and the iterative optimization of dedicated interventional devices, the efficiency of the dTRA is expected to be significantly improved, thus further narrowing the time gap with the TFA.

The TFA group showed a higher CNR value, which means that the signal difference between the target vessel (such as the inferior genicular artery) and the surrounding muscle and other tissues in this group was more significant. This enhanced contrast directly translates into higher vascular visibility, making the vascular edge more sharp, which is of great value in the diagnosis of distal vascular stenosis, calcification or occlusion. Although the femoral artery angiography can provide clearer images and better display the smaller distal vessels, but the angiography images obtained through the distal radial artery puncture are usually enough to meet our basic diagnostic needs for lesions below the knee. And in terms of postoperative braking management, dTRA demonstrates a unique advantage: a local compression bandage is required for only 2 h after surgery to achieve effective haemostasis, whereas a traditional femoral artery puncture requires a bedridden compression bandage for 6–8 h, a difference that significantly improves the patient's postoperative comfort and satisfaction with their visit to the doctor. In view of the characteristics of our department, the patients are mainly from the elderly diabetic foot group, which is often accompanied by lower limb ischemia and difficulty in wound healing, and there is an urgent need to accurately assess the blood supply of the lower limbs in order to formulate an individualized surgical plan. However, elderly patients are generally less tolerant of prolonged braking, and some patients with severe lower limb ischaemia suffer from severe pain, which further exacerbates physical discomfort during braking. In this context, with its minimally invasive access and rapid recovery, dTRA can not only accurately assess the blood supply of the lower limbs, but also provide a minimally invasive haemostatic method to provide both diagnostic value and humanistic care for the elderly high-risk patient group.

At last, there are several limitations in this study. The current generation of interventional devices has limited working lengths, which precluded the taller patients lead to selection bias. Nevertheless, this study still has significant clinical value and provides an evidence-based basis for the selection of access routes for lower limb arteriography. This result of this study showed comparable procedural success rates and complication profiles between the dTRA and TFA, but significant differences in contrast medium volume and total procedural time. Clinicians should consider the patient's specific situation, such as vascular conditions, underlying diseases, physical tolerance and other factors, to choose the most suitable puncture path for the patient, in order to achieve safe, efficient and accurate diagnosis of lower limb arteriography. Meanwhile, more large-sample, multicentre studies are required in the future to further validate and refine the conclusions of this study and to promote the development of the dTRA in lower limb arterial diagnosis and treatment.
